# Clinical complete response achieved in patients with breast cancer liver metastasis after personalized treatment: a case report and literature review

**DOI:** 10.3389/fonc.2025.1546691

**Published:** 2026-01-12

**Authors:** Yuxiang Wang, Na Jing, Yu Wang, Ning Li

**Affiliations:** 1 Ultrasound Department, Shanxi Province Cancer Hospital/Shanxi Hospital Affiliated to Cancer Hospital, Chinese Academy of Medical Sciences/Cancer Hospital Affiliated to Shanxi Medical University, Taiyuan, China; 2 Department of Radiotherapy, Shanxi Province Cancer Hospital/Shanxi Hospital Affiliated to Cancer Hospital, Chinese Academy of Medical Sciences/Cancer Hospital Affiliated to Shanxi Medical University, Taiyuan, China

**Keywords:** breast cancer, complete response, literature review, liver metastasis, patient-derived organoids

## Abstract

Patient-derived organoids (PDOs) have been demonstrated not only to predict the response to neoadjuvant chemotherapy but also to tailor the treatment options for advanced breast cancer to improve prognosis. Here, we first reported a case of breast cancer liver metastasis that obtained clinical complete response (cCR) after treatment with eribulin and capecitabine, which showed sensitivity in the organoid drug sensitivity testing, and we conducted a systematic literature review. Our case report further provides a scientific basis for the PDOs as a powerful preclinical model to predict individual treatment sensitivity and improve the treatment outcomes of patients with advanced breast cancer, especially those with refractory or heavily treated breast cancer.

## 1 Introduction

Breast cancer is the most frequent malignancy and the leading cause of cancer-related deaths in women worldwide (1). Its mortality is primarily ascribed to the complications caused by the development of distant metastases, with the liver, lung, and bone as the most common metastatic sites (2). At the time of diagnosis, approximately 5%–8% breast cancer patients develop distant metastases, among whom only 24%–39% survive for 5 years (3). It is estimated that there are 50%–70% of breast cancer patients with liver metastasis (4). After metastasis to the liver, the prognosis of patients is usually poor, with a median survival of 2–3 years (5). Recently, therapeutic advancements have significantly improved the survival outcomes of breast cancer patients, which is partially attributed to the progress in endocrine and targeted treatment strategies, particularly the introduction of cyclin-dependent kinase 4/6 inhibitors (6). However, intra- and inter-tumor heterogeneity often results in significant variations in treatment efficacy and the emergence of drug resistance (7). Therefore, it is very necessary to seek a preclinical model that can predict the individualized drug response to optimize the treatment regimens.

Conventional cancer cell lines are easy to propagate but are unable to maintain the three-dimensional (3D) structures and the patient heterogeneity (8). Although patient-derived xenografts can preserve the biological and molecular features and the tumor heterogeneity of the original tumor, they are expensive, labor-intensive, and time-consuming (9). Unlike them, patient-derived organoids (PDOs), a promising 3D preclinical model, have been established in multiple cancer types, such as breast cancer, pancreatic cancer, and colorectal cancer, due to faithful recapitulation of the tumor characteristics at genomic, transcriptomic, and proteomic levels (10–12). PDOs are closer to the actual tumor microenvironment under the 3D context and allow for biomarker discovery and drug screening to identify potential drugs at the individual level (13). In previous studies on breast cancer, PDOs have been demonstrated not only to predict the response to neoadjuvant chemotherapy but also to tailor the treatment options for advanced patients to improve prognosis (10, 14). Here, we first reported a typical case of breast cancer liver metastasis that obtained clinical complete response (cCR) after treatment with drugs identified by the PDOs.

## 2 Case presentation

A 30-year-old woman was admitted to the hospital on May 24, 2024, with the chief complaint of breast cancer liver metastasis for over 6 months occurring more than 4 years after breast-conserving surgery for right breast cancer.

**Figure 1:**
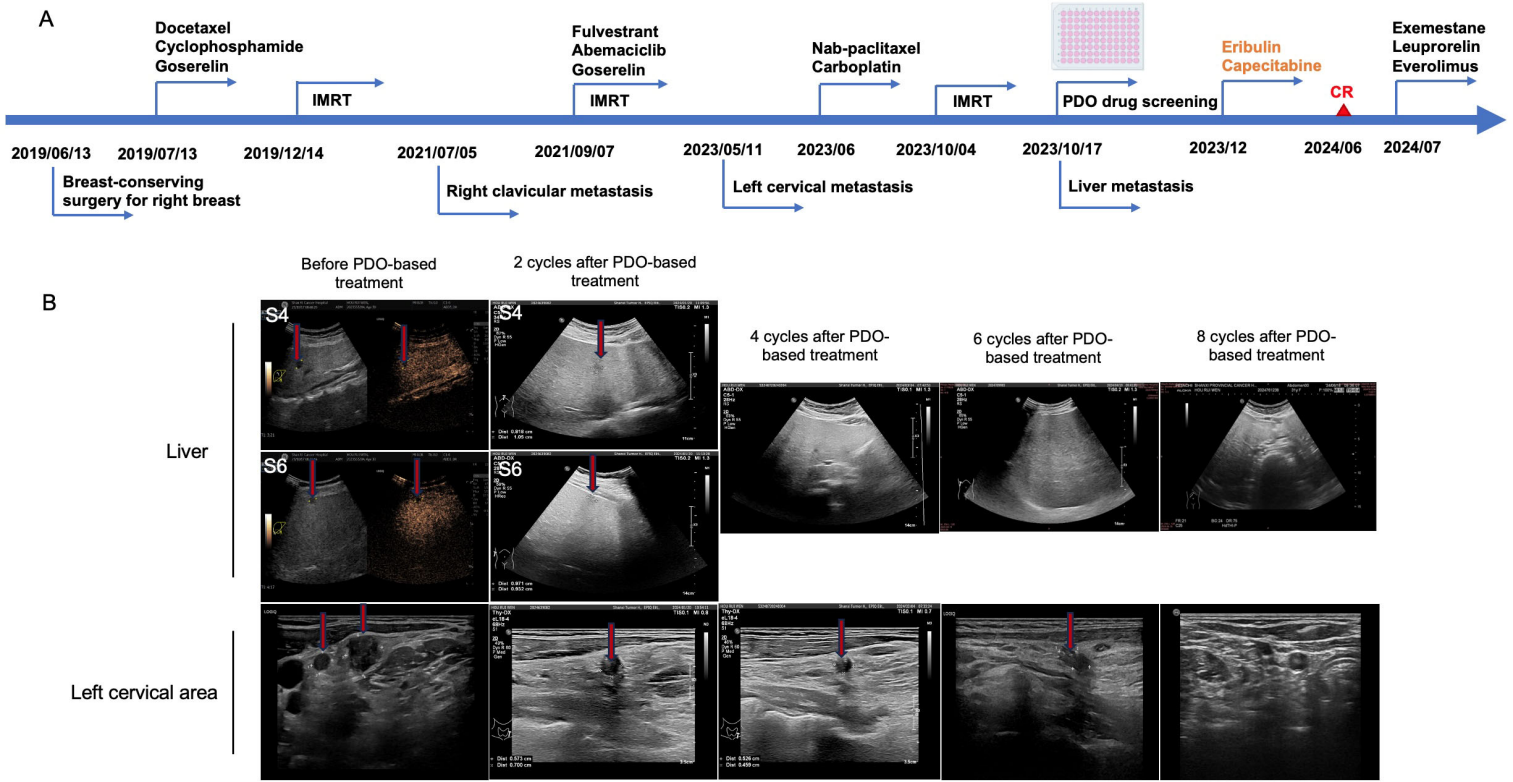
Advanced breast cancer with left cervical lymphadenectasis and liver metastasis. **(A)** The clinical treatment process of the patient. **(B)** Representative ultrasound images of the liver and left cervical area before patient-derived organoid (PDO)-based treatment, and after two, four, six, and eight cycles of PDO-based treatment. The red arrows point to the lesions. It could be observed that there were no significant space-occupying lesions in the liver after 4 cycles; the number and size of lymphadenectasis in the left cervical area IV were significantly reduced after two cycles and disappeared after eight cycles.

On June 13, 2019, the patient underwent breast-conserving surgery for right breast cancer (**Figure 1A**). Postoperative pathology showed grade III invasive ductal carcinoma with a tumor size of 1.3 cm, with multifocal high-intermediate ductal carcinoma *in situ*. There were no clear vascular cancer thrombi or nerve invasion, and no cancer cells at the margins. Sentinel lymph node metastasis was 0/5. Immunohistochemistry (IHC) indicated estrogen receptors (ER)-positive (70%–80%), progesterone receptor (PR)-positive (90%), human epidermal growth factor receptor 2 (HER2)-negative, and Ki67 of 40%–50%. The patient was diagnosed with pT1cN0M0, stage IA, without mutations in the *BRCA* genes. On July 13, 2019, docetaxel and cyclophosphamide as adjuvant chemotherapy were applied for four cycles, during which goserelin was used for ovarian function suppression. After chemotherapy, the patient started to take tamoxifen orally. Between December 24, 2019, and January 13, 2020, intensity-modulated radiation therapy (IMRT) was performed on the right breast (43.5 Gy/2.9 Gy/15 f) and tumor bed (49.5 Gy/3.3 Gy/15 f) with 6-MV X-ray. On July 5, 2021, ultrasound examinations showed lymphadenectasis in the right clavicular area. Core-needle biopsy indicated poorly differentiated carcinoma. Additionally, IHC showed ER (90%+), PR (10%+), HER2 (1+), Ki-67 (70%+), CK5 (−), E-cadherin (−), AE1/AE3 (+), CK7 (+), GATA3 (+), TTF-1 (−), and Napsin-A (−). Metastatic breast cancer was considered. On September 7, 2021, she underwent IMRT for right subclavicular and supraclavicular regions (50 Gy/2.0 Gy/25 f) and for lymphadenectasis in the right clavicular region (60 Gy/2.4 Gy/25 f) with 6-MV X-ray. After completing IMRT, the patient received the treatment of fulvestrant, abemaciclib, and goserelin until May 2023.

On May 11, 2023, ultrasound examinations showed lymphadenectasis of less than 1 cm in the left cervical area IV; thus, core-needle biopsy for the left cervical lymph nodes was performed, suggesting adenocarcinoma cells. Through IHC, ER (small amount +), PR (−), HER2 (−), and Ki67 (individual +) were observed. In June 2023, the patient was treated with six cycles of nab-paclitaxel (400 mg) and carboplatin (400 mg) as salvage chemotherapy. On October 4, 2023, palliative radiotherapy was performed, including IMRT for the left subclavicular and supraclavicular regions (50 Gy/2.0 Gy/25 f) and IMRT for lymphadenectasis in the left clavicular region (60 Gy/2.4 Gy/30 f) with 6-MV X-ray. On October 17, 2023, liver contrast-enhanced ultrasonography showed multiple new nodules in the liver (1.38 × 1.09 cm in the left lobe and 0.92 × 0.53 cm in the right lobe; **Figure 1B**), suggesting metastasis. One week later, a liver biopsy was conducted. In combination with medical history and IHC results [ER (80%+), PR (−), HER2 (1+), Ki-67 (80%+), CK5 (−), hepatocyte (−), and GATA3 (+)], metastatic carcinoma was diagnosed, suggestive of breast origin.

**Figure 2:**
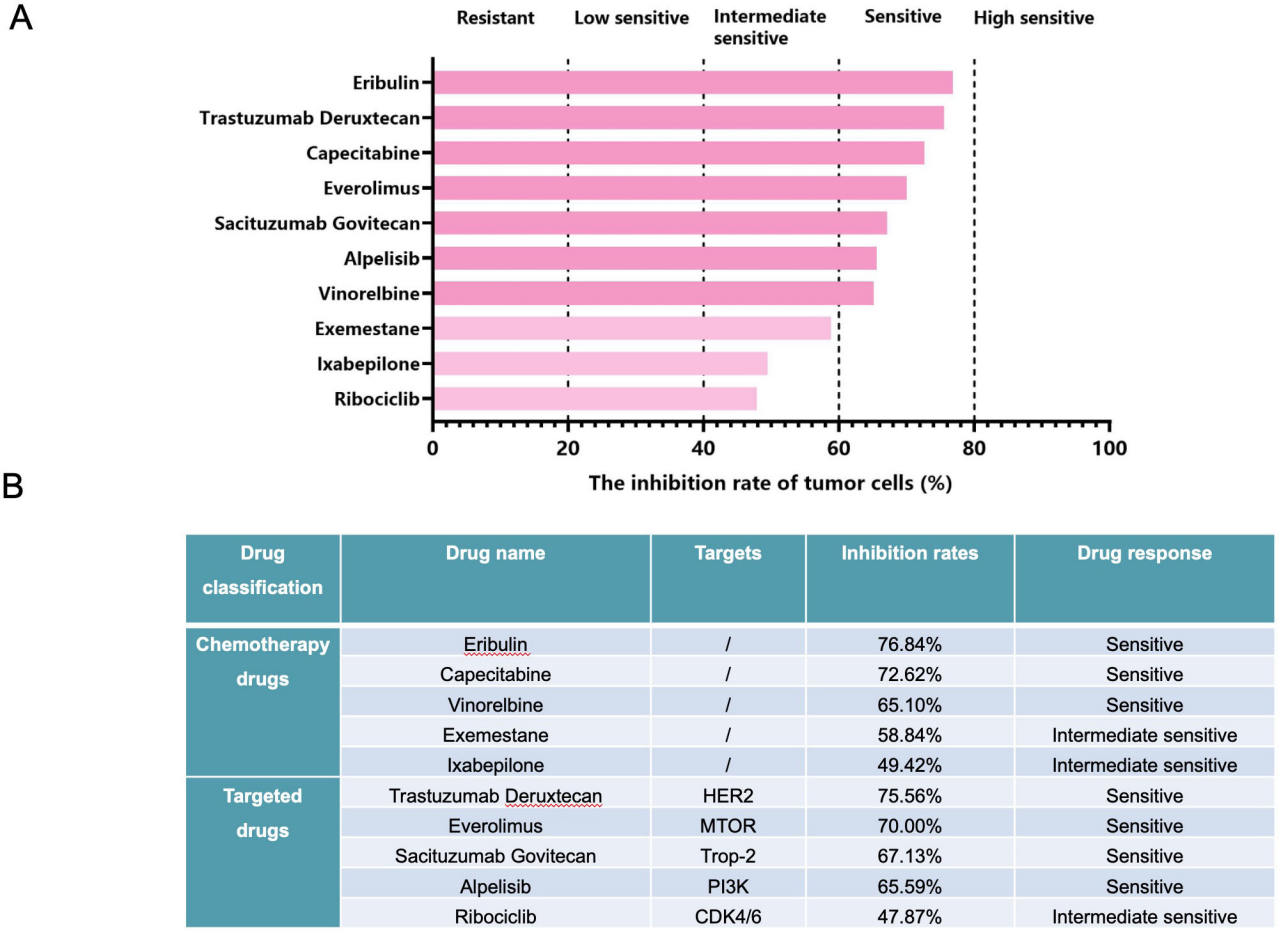
The results of drug sensitivity testing based on the breast cancer organoids from liver biopsy **(A)** and the drug panel used for drug sensitivity testing and their corresponding inhibition rates against tumor cells **(B)**.

After informed consent was obtained, the fresh biopsy tissue from the liver lesion was collected for organoid culture performed in the laboratory of Kingbio Medical Co., Ltd., Chongqing, China. The sample was first washed with precooled phosphate-buffered saline and then cut into pieces. After digestion for 30 min, the cell pellet was obtained through centrifugation. Subsequently, Matrigel was added, and cells and Matrigel suspension were both seeded onto six-well plates (2 mL per well) using pipettes, which were placed in a 37°C incubator for 15 min. When the droplets were formed, an appropriate amount of Jiabili^®^ culture medium was added, which was then cultured at 37°C, 5% CO_2_. The culture medium was replaced every 2–3 days. The status of organoid growth was observed under optical microscopes. When organoids were like solid spheroids with approximately 70 µm in diameter, drug sensitivity testing was performed. As shown in the drug sensitivity testing, eribulin, trastuzumab deruxtecan, capecitabine, and everolimus may be the potential candidates for this patient (**Figure 2**). Accordingly, in December 2023, the patient was treated with eribulin (2 mg, d1, 8) and capecitabine (1,000 mg/m^2^, bid, d1–14) due to trastuzumab deruxtecan’s high cost, during which the therapeutic effect was assessed every two cycles based on ultrasound examinations. It could be observed that after eight cycles of treatment, there were no significant space-occupying lesions in the liver, and the number and size of lymphadenectasis in the left cervical area IV were gradually decreased until no lymph nodes were present (**Figure 1B**), suggesting that the patient achieved cCR in both the left cervical area and the liver. At present, chemotherapy is discontinued due to neurotoxicity, and endocrine therapy with exemestane, leuprorelin, and everolimus is used.

## 3 Discussion

Resistance to treatment has been one of the leading challenges for advanced breast cancer. The latent mechanisms of treatment resistance are thought to be associated with genomic alterations, variations in tumor microenvironment, and activation of multiple signaling pathways (15, 16), providing some insights into the possible effects of drug therapies on the genomic mutations in breast cancer to a certain extent. Nevertheless, there is little information used for the prediction of cancer treatment outcomes and patient stratification. The PDOs, a promising preclinical model faithfully preserving the biological and molecular features of advanced breast cancer and treatment response, have been confirmed to guide the clinical treatment of advanced breast cancer (10). In this study, a breast cancer patient with contralateral cervical lymphadenectasis and liver metastasis finally achieved cCR, whether in the left cervical area or in the liver, after the treatment guided by the PDO-based drug screening, further highlighting the great potential of PDOs as a real-time platform in tailoring drug therapies for advanced breast cancer.

Currently, organoid technology has been gradually applied in multiple cancer types, but whether the PDOs from heavily treated tumor samples can be cultured successfully needs to be further investigated. Under normal circumstances, cancer patients present resistance to a large range of drugs following failure in various therapies, consequently leading to an urgent need for personalized treatment. The PDOs, a three-dimensional cell culture system, represent a promising model for precision medicine in cancer due to accurate recapitulation of the important characteristics of the original tumors, such as genetic and cellular heterogeneity. They can not only reflect the efficacy of previous treatments but also predict the treatment sensitivity at the patient-specific level (17). In previously reported cases, the PDO models were established for guiding the treatment options for breast cancer patients with different molecular types, and the results showed that, except for one case with stable disease, all other cases obtained effective tumor regression after the treatment with sensitive drugs through PDO-based drug sensitivity testing (**Table 1**). Notably, in our study, the patient still developed metastases, although multiple standard treatments were used. However, when eribulin and capecitabine, which were shown to be sensitive by organoid drug sensitivity testing, were applied for eight cycles, lymphadenectasis in the left cervical area and space-occupying lesions in the liver both disappeared, suggesting cCR was achieved in both lesions. These data further supported that the PDOs could be cultured successfully in patients with heavily treated breast cancer and could be a promising real-time *in vitro* model to tailor the clinical treatment options for advanced breast cancer.

**Table 1:** The baseline characteristics and treatment outcomes of breast cancer patients guided by the PDO-based drug sensitivity testing.

No.	Disease types	Previous treatment	Previous surgery	Disease condition	Sensitive drugs based on the PDOs	Treatment	Outcomes
Case 1 (11)	Advanced breast cancer	Five rounds of treatment	No	Multiple lung metastases and resistance to 5 lines of treatment	Doxorubicin, everolimus, and epirubicin	Lipo‐doxorubicin, everolimus, and bevacizumab	Effective tumor regression
Case 2 (11)	TNBC	Neoadjuvant chemotherapy with epirubicin, cyclophosphamide, docetaxel, and carboplatin	Yes	Multiple lung metastases and resistance to capecitabine	Everolimus, mitoxantrone, and vinorelbine	Everolimus and vinorelbine	Stable disease
Case 3 (11)	TNBC	Neoadjuvant chemotherapy with doxorubicin, cyclophosphamide, docetaxel, and carboplatin	Yes	Liver metastasis after 1 cycle of nab-paclitaxel	Temsirolimus, neratinib, and bortezomib	Bortezomib	Tumor reduction in the liver
Case 4 (in our study)	Invasive ductal carcinoma	Adjuvant chemotherapy with pirarubicin and cyclophosphamide, goserelin plus IMRT	Yes	Liver metastasis, secondary to cervical and clavicular lymphadenectasis	Eribulin, trastuzumab deruxtecan, capecitabine, and everolimus	Eribulin and capecitabine	Clinical complete response

TNBC, triple-negative breast cancer; PDO, patient-derived organoid.

Eribulin, a novel microtubule dynamics inhibitor, is distinct from other conventional tubulin-targeting agents. It can induce vascular remodeling, which may improve tumor perfusion, leading to increased intratumoral drug penetration and potentially enhanced efficacy (18). Currently, eribulin is used for treating locally advanced or metastatic breast cancer patients who have progressed after the second- or third-line taxane or anthracycline-based regimens. Capecitabine, a fluoropyrimidine carbamate prodrug of 5′-fluorouracil, is also commonly used for treating metastatic breast cancer patients whose disease is resistant to taxane or anthracycline or both. Interestingly, additive efficacy was shown in xenograft models when eribulin was utilized in combination with capecitabine (19). In a phase 1b/2, open-label study, the combination of eribulin and capecitabine showed promising efficacy in metastatic breast cancer patients, with manageable tolerability (20). Our findings further support the feasibility of eribulin combined with capecitabine in the treatment of metastatic breast cancer.

There are several limitations in our study. First, this is a case report. The efficacy of PDO-guided treatment observed in this case may not be generalized to all advanced breast cancer patients with liver metastasis, as tumor heterogeneity can lead to variable responses to PDO-based drug screening. Second, the long-term follow-up data are absent, which may limit the ability of PDOs to guide long-term survival outcomes. Third, the genetic correlation between PDOs and the patient’s primary/metastatic tumor was not analyzed in this study, likely affecting the interpretation of PDO-guided drug screening results. Despite the above limitations, PDOs hold substantial promise for optimizing personalized treatment of advanced breast cancer. In future studies, integrating PDOs with multi-omics technologies, such as whole-exome sequencing, transcriptomics, and metabolomics, will enable comprehensive characterization of the molecular landscape of PDOs and their matching tumors. This will help clarify the molecular mechanisms underlying PDO drug response consistency or discrepancy and identify potential predictive biomarkers for treatment efficacy. In addition, a combination of PDO-based drug screening with artificial intelligence (AI)-driven platforms offers new opportunities for cancer research. AI algorithms can analyze large-scale data from PDO drug sensitivity testing, multi-omics profiles, and clinical outcomes to establish predictive models for patient-specific drug responses. Such models may significantly improve the efficiency and accuracy of PDO-guided treatment decision-making, helping to rapidly prioritize the most effective treatment regimens for patients with advanced or metastatic breast cancer.

In conclusion, we first described a case of breast cancer liver metastasis that achieved cCR based on the personalized treatment guided by the organoid drug sensitivity testing. This case report further provides a scientific basis for the PDOs as a powerful preclinical model to predict individual treatment sensitivity and improve treatment outcomes in patients with advanced breast cancer, especially for those with refractory or heavily treated breast cancer.

## Data Availability

The original contributions presented in the study are included in the article/supplementary material. Further inquiries can be directed to the corresponding authors.
